# Tumor-Infiltrating Myeloid Cells Co-Express TREM1 and TREM2 and Elevated TREM-1 Associates With Disease Progression in Renal Cell Carcinoma

**DOI:** 10.3389/fonc.2021.662723

**Published:** 2022-02-10

**Authors:** Jill W. Ford, Marieli Gonzalez-Cotto, Alexander W. MacFarlane, Suraj Peri, O. M. Zack Howard, Jeffrey J. Subleski, Karen J. Ruth, Mohammed Haseebuddin, Tahseen Al-Saleem, Youfeng Yang, Pat Rayman, Brian Rini, W. Marston Linehan, James Finke, Jonathan M. Weiss, Kerry S. Campbell, Daniel W. McVicar

**Affiliations:** ^1^ Laboratory of Cancer Immunometabolism, National Cancer Institute (NCI), Frederick, MD, United States; ^2^ Blood Cell Development and Function Program, Institute for Cancer Research, Philadelphia, PA, United States; ^3^ Biostatistics and Bioinformatics Facility, Fox Chase Cancer Center, Philadelphia, PA, United States; ^4^ Department of Surgical Oncology, Fox Chase Cancer Center, Philadelphia, PA, United States; ^5^ Department of Pathology, Fox Chase Cancer Center, Philadelphia, PA, United States; ^6^ Urologic Oncology Branch, National Cancer Institute (NCI), Bethesda, MD, United States; ^7^ Cleveland Clinic, Department of Immunology, Lerner Research Institute, Cleveland, OH, United States; ^8^ Cleveland Clinic, Department of Solid Tumor Oncology, Cleveland, OH, United States

**Keywords:** TREM family, renal cell carcinoma, myeloid-derived suppressor cell, tumor-associated macrophage, sTREM-1, TREM2 (triggering receptor expressed on myeloid cells), TREM1

## Abstract

Myeloid-derived suppressor cells (MDSC) and tumor-associated macrophages (TAM) contribute to cancer-related inflammation and tumor progression. While several myeloid molecules have been ascribed a regulatory function in these processes, the triggering receptors expressed on myeloid cells (TREMs) have emerged as potent modulators of the innate immune response. While various TREMs amplify inflammation, others dampen it and are emerging as important players in modulating tumor progression—for instance, soluble TREM-1 (sTREM-1), which is detected during inflammation, associates with disease progression, while TREM-2 expression is associated with tumor-promoting macrophages. We hypothesized that TREM-1 and TREM-2 might be co-expressed on tumor-infiltrating myeloid cells and that elevated sTREM-1 associates with disease outcomes, thus representing a possibility for mutual modulation in cancer. Using the 4T1 breast cancer model, we found TREM-1 and TREM-2 expression on MDSC and TAM and that sTREM-1 was elevated in tumor-bearing mice in multiple models and correlated with tumor volume. While TREM-1 engagement enhanced TNF, a TREM-2 ligand was detected on MDSC and TAM, suggesting that both TREM could be functional in the tumor setting. Similarly, we detected TREM-1 and *Trem2* expression in myeloid cells in the RENCA model of renal cell carcinoma (RCC). We confirmed these findings in human disease by demonstrating the expression of TREM-1 on tumor-infiltrating myeloid cells from patients with RCC and finding that sTREM-1 was increased in patients with RCC. Finally, The Cancer Genome Atlas analysis shows that *TREM1* expression in tumors correlates with poor outcomes in RCC. Taken together, our data suggest that manipulation of the TREM-1/TREM-2 balance in tumors may be a novel means to modulate tumor-infiltrating myeloid cell phenotype and function.

## Introduction

The Triggering Receptor Expressed on Myeloid cells (TREM) and TREM-like (TLT) proteins are a family of cell surface receptors expressed on granulocytes, monocytes, macrophages, microglia, dendritic cells (DC), osteoclasts, and platelets (1). The presence of at least one TREM on virtually every myeloid cell population provides them with a unique ability to regulate inflammation. Accordingly, TREM have been linked to many diseases, including sepsis, inflammatory bowel disease, Nasu–Hakola disease, osteopetrosis, and multiple sclerosis (MS) (1, 2). Of the TREM and TLT, TREM-1, TREM-2, and TLT-1 are best characterized.

TREM-1 is expressed on neutrophils and monocytes and amplifies inflammatory responses by synergizing with pattern recognition receptors (PRR) to induce pro-inflammatory mediators, such as IL-8, TNF, and MCP-1 (3). Blockade of TREM-1 with TREM-1/Fc fusion protein, TREM-1 peptide, or TREM-1 siRNA rescues mice from sepsis (4–6) and delays tumor progression in xenographs (7). In contrast to TREM-1, TREM-2 is expressed on macrophages, microglia, DC, and osteoclasts and is thought to be anti-inflammatory by virtue of its *cis* interaction with a co-expressed TREM-2 ligand (8–10). Macrophages derived from the bone marrow of *Trem2^-/-^* mice produce increased TNF and IL-6 after stimulation with TLR agonists (9). Additional evidence for the dampening roles of TREM-2 comes from recent studies in which TREM-2 was shown to suppress EAE, the mouse model of MS (11, 12), and to promote colonic wound healing through increased production of the anti-inflammatory cytokines IL-4 and IL-13 in the wound bed (13).

A soluble form of TREM-1 (sTREM-1) has been detected in patients and animals suffering from various inflammatory conditions and often correlates with disease severity (2). Most notably, sTREM-1 is elevated in serum and plasma during sepsis (14–16). Recently, soluble TREM-2 (sTREM-2) was found in the cerebrospinal fluid of patients with MS and other inflammatory neurological diseases (17). Although the functions of TREM within the tumor microenvironment are incompletely understood, it is possible that heterologous interactions in *cis* or *trans* may control TREM signaling, thus modulating inflammatory outcomes.

In healthy individuals, TREM-1 is present on CD14^hi^ or “classical” monocytes, while it is absent from CD14^dim^ monocytes (3, 18). Similar to humans, two populations of monocytes, resident and inflammatory, are present in mice. The resident monocytes are Gr-1(Ly6C)^-^CD11b^+^F4/80^+^ and are thought to replenish tissue macrophages, while the inflammatory monocytes are Gr-1(Ly6C)^+^CD11b^+^F4/80^+^ and are rapidly recruited to sites of inflammation (19). While TREM-1 is constitutively expressed on resident monocytes, it is absent from inflammatory monocytes but can be upregulated on them after the lipopolysaccharide (LPS) challenge (18, 20).

Mouse inflammatory monocytes resemble a population of cells sometimes referred to as myeloid-derived suppressor cells (MDSC). MDSC are found in the blood of patients with many types of cancer and in the bone marrow, blood, spleen, and tumors of tumor-bearing mice (21). MDSC are a heterogeneous mixture of myeloid cells that suppress T cell responses and contribute to tumor-associated inflammation and tumor progression (22, 23). In murine cancer models, two types of MDSC have been observed. Granulocytic MDSC (PMN-MDSC) are phenotypically similar to neutrophils and are characterized as Gr-1(Ly6G)^hi^CD11b^+^F4/80^-^, while monocytic MDSC (Mo-MDSC) are phenotypically similar to inflammatory monocytes and are characterized as Gr-1(Ly6C)^+^CD11b^+^F4/80^+^ (24, 25). Although the phenotype of human MDSC has been more difficult to characterize, most are defined as either HLADR^-^ CD11b^+^ CD14^-^ (CD15 or CD66b)^+^ CD33^dim^ for the PMN-MDSC or CD11b^+^ CD14^+^ HLADR^dim/-^ (CD15 or CD66b)^-^ CD33^+^ for M-MDSC (26, 27)

We hypothesized that TREM-1 would be overexpressed on MDSC in tumor-bearing mice and that TREM-2 would be co-expressed on MDSC and TAM in tumor-bearing animals. Thus, this balance of pro-inflammatory TREM-1 to anti-inflammatory TREM-2 might, in part, control the phenotype and function of tumor-infiltrating myeloid cells. In this study, we found that both TREM-1 and TREM-2 were expressed on MDSC and TAM and that tumor-bearing mice have elevated levels of sTREM-1 in their blood. Additionally, we demonstrated the presence of a TREM-2 ligand on TAM and showed that TREM-1 on MDSC was functional, suggesting that both TREMs may regulate these myeloid populations within the tumor, thus possibly balancing each other. We confirmed the significance of our findings by demonstrating TREM-1 expression on tumor-infiltrating monocytes and neutrophils in renal cell carcinoma (RCC) donors. We also detected elevated levels of sTREM-1 in patients suffering from RCC, suggesting the potential use of sTREM-1 as a biomarker for tumor progression. Furthermore, using The Cancer Genome Atlas (TCGA) database, we found that, although not an individual predictor for survival, TREM-1 expression correlates with disease severity and tumor burden. Thus, we propose that TREM-1 may serve as a biomarker for tumor progression and a useful target for reprogramming the immunosuppressive tumor microenvironment of the host and that TREM2 might play an important role in balancing the inflammatory signals within the tumor.

## Results

### Trem-1 Is Expressed in MDSCs and TAMs of Tumor-Bearing Mice

**Figure 1:**
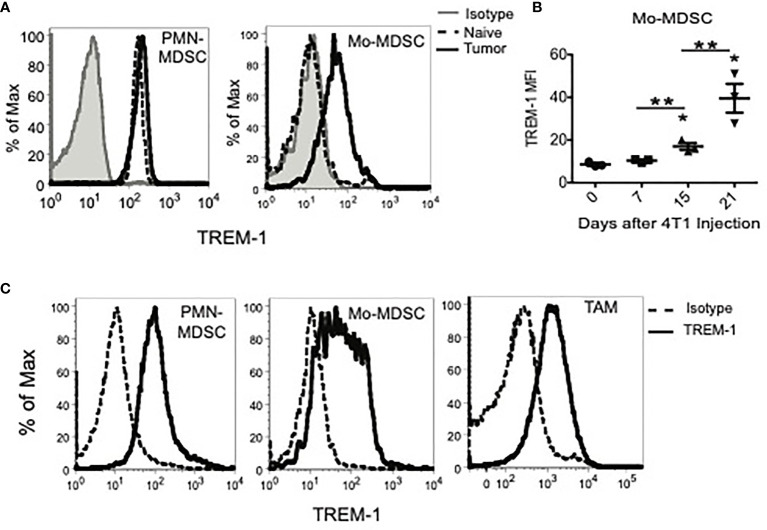
TREM-1 is expressed on peripheral myeloid-derived suppressor cells (MDSC) subsets, tumor-infiltrating MDSC, and tumor-associated macrophages in tumor-bearing mice. **(A)** TREM-1 expression on splenic granulocytic MDSC (PMN-MDSC) and monocytic MDSC (Mo-MDSC) from naïve or tumor-bearing mice 21 days after 4T1 inoculation. **(B)** TREM-1 levels on tumor-derived Mo-MDSC 0, 7, 15, and 21 days after the 4T1 tumor challenge. *p < 0.05 relative to naïve mice, **p < 0.0. **(C)** TREM-1 expression on PMN-MDSC, Mo-MDSC, and tumor-associated macrophages isolated from tumors of tumor-bearing mice 21 days after 4T1 injection. One representative histogram is shown for each population out of more than three independent experiments.

In order to determine the TREM expression profile in the tumor context, we initially utilized the 4T1 murine breast carcinoma model, which induces large numbers of MDSC in mice (**Supplementary Figure S1**) (28, 29). We found, by flow cytometry, that TREM-1 was expressed on PMN-MDSC and Mo-MDSC in the blood and spleens of tumor-bearing mice (**Figure 1A** and data not shown) regardless of the approach that we used to identify MDSC (**Supplementary Figures S2** and **S3**). While TREM-1 levels were only slightly increased on PMN-MDSC relative to PMN of naïve mice, TREM-1 levels on Mo-MDSC increased significantly with time after 4T1 injection (**Figure 1B**). TREM-1 was not expressed on the phenotypically similar counterpart (inflammatory monocytes) of Mo-MDSC in naïve animals (3). The expression of TREM-1 on MDSC was not restricted to the 4T1 model or the BALB/c strain as TREM-1 was also expressed on PMN- and Mo-MDSC from EL-4 tumor-bearing C57BL/6 mice (data not shown).

As the 4T1 tumor volume increases *in vivo*, the percentage of myeloid cells infiltrating the tumor also increases and is comprised mainly of PMN-MDSC, Mo-MDSC, and TAM (**Supplementary Figure S3**). Therefore, we examined TREM-1 expression on MDSC and TAM present in tumors of 4T1, EL-4, and MC38 tumor-bearing mice (**Figure 1C** and data not shown). Consistent with our mRNA analysis, TREM-1 was expressed on tumor-infiltrating PMN-MDSC, Mo-MDSC, and TAM in all three tumor models. TREM-1 expression on Mo-MDSC was more heterogeneous than that on PMN-MDSC and TAM, with some cells expressing low or no TREM-1 and others expressing high TREM-1, suggesting that Mo-MDSC represent a more diverse population of cells than PMN-MDSC or TAM. Overall, TREM-1 is expressed in tumor-infiltrating MDSC and is notably upregulated in PMN-MDSC during tumor progression.

### Expression of Trem-1 in MDSC Is Functional and Trem1-Expressing MDSC Suppress T Cell Proliferation

**Figure 2:**
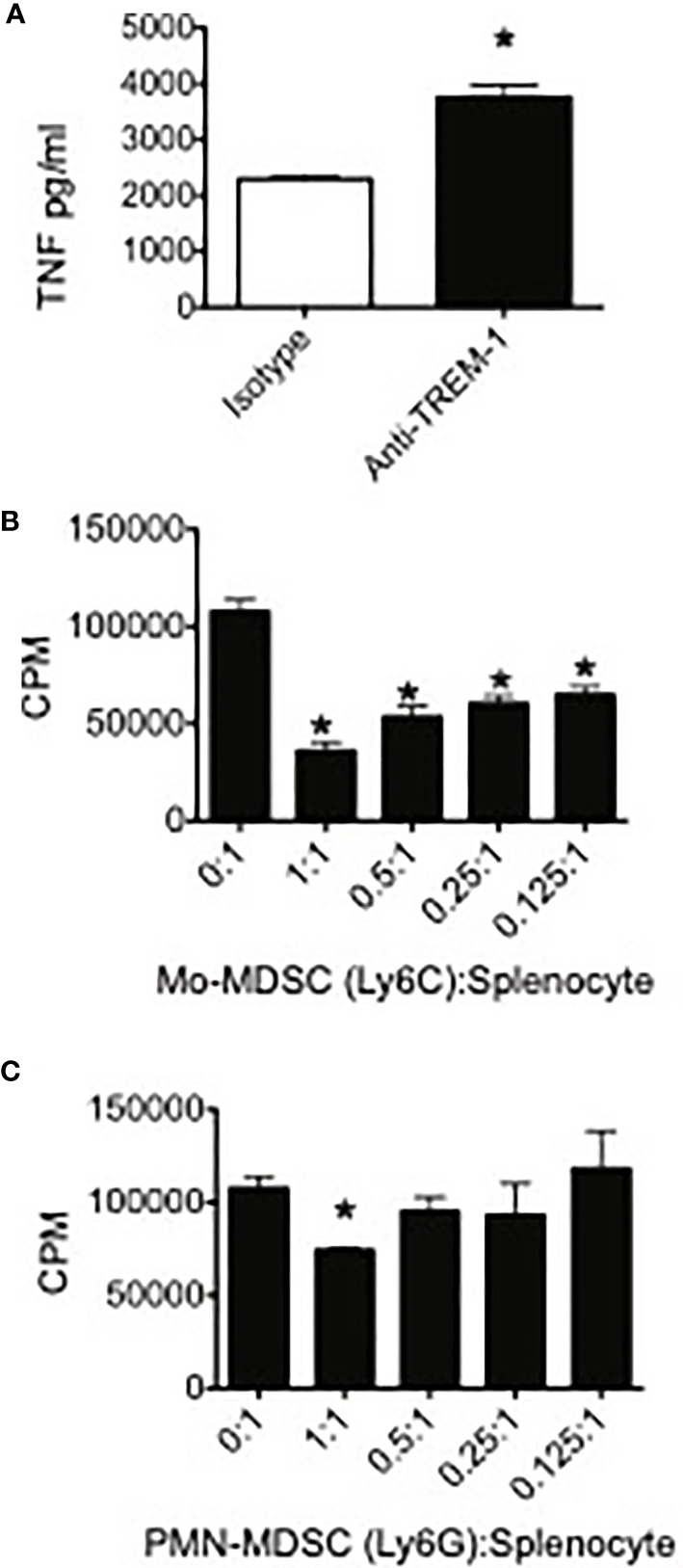
TREM-1 engagement enhances pro-inflammatory cytokine release by myeloid-derived suppressor cells (MDSC) and Trem-1^+^ MDSC from tumor-bearing mice suppress T cell proliferation. **(A)** Peripheral myeloid-derived suppressor cells were sorted from spleens of 4T1 tumor-bearing mice, plated onto wells coated with either anti-TREM-1 or rat IgG2A isotype control Ab, and stimulated with lipopolysaccharide. The supernatants were harvested 20 h later, and the TNF levels in the supernatants were measured by ELISA. *p < 0.05. **(B)** Ly6C^+^CD11b^+^ (Mo) and **(C)** Ly6G^+^CD11b^+^ (PMN) MDSC were sorted from spleens of 4T1 tumor-bearing mice. The cells were cultured at the indicated ratios with splenocytes from naïve BALB/c mice. Con A was added to each well at a final concentration of 2 μg/ml. Tritiated thymidine incorporation was measured during the last 18 h of the 85-h incubation period. *p-value <0.05 relative to no suppressors. Data represents mean ± SEM from triplicates of a representative experiment.

In order to determine if TREM-1 expressed on MDSC was functional, we used TNF production as a marker for TREM-1-mediated activation. Because the identity of the TREM-1 ligand(s) remains largely unknown, we used anti-TREM-1 to engage the receptor on the surface of PMN-MDSC sorted from spleens of 4T1 tumor-bearing mice. Compared to isotype control-stimulated cells, TREM-1 engagement resulted in enhanced LPS-induced TNF release, suggesting that TREM-1 on MDSC, and presumably TAM, is functional and may play a role in increasing pro-inflammatory cytokines from these cells while decreasing immunosuppressive factors (**Figure 2A**).

One of the hallmarks of MDSC is their ability to inhibit T cell proliferative responses. In order to demonstrate that TREM-1^+^ PMN-MDSC and Mo-MDSC were suppressive, we sorted these populations from spleens of 4T1-injected mice and measured their capacity to inhibit Con A-induced T cell proliferation. Both TREM-1^+^ PMN-MDSC and TREM-1^+^ Mo-MDSC suppressed T cell proliferation (**Figures 2B, C**). In accordance with our previous results demonstrating increased TREM-1 expression in Mo-MDSC during tumor progression, we found that Mo-MDSC were more suppressive than PMN-MDSC, inhibiting Con A-induced T cell proliferation at lower MDSC-to-splenocyte ratios.

### TREM-2 Is Expressed in Mo-MDSC and TAM

As TAM are frequently described as type 2 or alternatively activated macrophages that facilitate tumor growth, we hypothesized that a counteracting TREM receptor, such as TREM-2, might be expressed on these cells since this TREM is generally considered anti-inflammatory, has been shown to be expressed on alternatively activated macrophages, and has been associated with a type 2 macrophage gene signature (9, 30, 31).

Using FACS with multiple anti-TREM-2 antibodies, we were unable to detect TREM-2 on MDSC or TAM in our models. Since detection of TREM-2 *ex vivo* is limited (9), the tumor microenvironment may have prevented us from detecting TREM-2, possibly through masking or cleavage of the receptor. As an alternative approach to FACS, we examined the plasma and tumor washes for sTREM-2 by ELISA. We detected substantial

**Figure 3:**
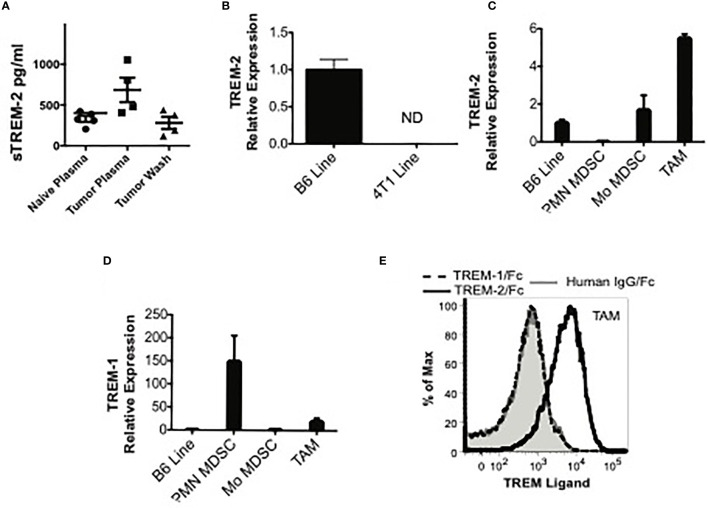
TREM-2 is expressed in monocytic MDSC (Mo-MDSC) and tumor-associated macrophages (TAM), and a possible ligand is expressed in TAM. **(A)** sTREM-2 levels in the plasma of naïve, 4TI-bearing mice or in the wash of 4T1 excised tumors. **(B, C)** *Trem2* expression by qPCR in B6 macrophages, 4T1 cells, splenic MDSC, or TAM as indicated. **(B)** Relative *Trem2* mRNA expression in B6 macrophages compared to 4TI cells demonstrating a neglectable *Trem1* expression in tumor cells. **(C, D)** Relative *Trem2* and *Trem1* mRNA expression in sorted splenic PMN-MDSC, Mo-MDSC, or TAM sorted from 4T1 tumors removed from mice (see **Supplementary Figures S2**–**S4** for the gating strategy). Data are normalized to a *Trem1*- and *Trem2*-expressing macrophage cell line (B6 Line). **(E)** Single-cell suspensions from 4T1 tumors from tumor-bearing mice were stained with MDSC markers and human (hu) IgG-Fc (gray shaded), TREM-1/hu IgG-Fc (dashed line), or TREM-2/hu IgG-Fc (solid line, no shading), followed by PE-labeled anti-human IgG-Fc. The TREM/hu IgG-Fc expression is shown on gated Gr-1^-^CD11b^+^ TAM.

sTREM-2 in the plasma of healthy mice, somewhat increased sTREM-2 in the plasma of tumor-bearing mice, and sTREM-2 in tumor interstitial fluid (**Figure 3A**). To rule out the tumor itself as a possible supply of sTREM-2, we examined *Trem2* message levels in 4T1 tumor cells. We detected no *Trem2* mRNA in 4T1 tumor cells when compared to a macrophage line (**Figure 3B**). Thus, the tumor cells themselves provide a negligible contribution, if any, to the Trem-2 and sTrem-2 present in tumor-bearing mice. In order to determine if sTrem-2 was being processed and/or shed from TAM, we sorted TAM from the tumors of 4T1-challenged mice and analyzed them for *Trem2* and *Trem1* mRNA levels. As shown in **Figures 3C, D**, TAM expressed 5.5 times more *Trem2* message and 19 times more *Trem1* mRNA than a B6 macrophage line known to express the receptors. In addition to TAM, we examined the *Trem2* and *Trem1* message levels in Mo-MDSC and PMN-MDSC sorted from spleens of 4T1-bearing mice. While *Trem2* message levels were not as high in Mo-MDSC as they were in TAM, they were increased relative to those of the macrophage line (**Figure 1C**). *Trem2* mRNA was not detected in PMN-MDSC even though *Trem1* mRNA was very high in this population. Thus, TAM and Mo-MDSC are the most likely sources of *Trem2* within the tumor, whereas PMN-MDSC and TAM are the primary sources of *Trem1.*

### A Ligand for Trem-2, But Not Trem-1, Is Present on MDSC and TAM

The presence of a TREM-1 or TREM-2 ligand within the tumor milieu would suggest that TREM could directly regulate TAM and/or MDSC at the tumor site. Although the identity of the TREM ligand(s) remains incompletely defined, using TREM-1/Fc chimera, an inducible ligand has been detected on the surface of neutrophils from LPS-challenged mice and on human platelets (5, 20, 32). Using a similar methodology, a TREM-2 ligand has been detected on bone marrow-derived and peritoneal macrophages, astrocytes, and osteoblasts (8, 11, 33, 34). While the astrocyte ligand has been identified as Hsp60, the identity of the ligand expressed by macrophages and osteoblasts is still unknown (35). Therefore, we examined the expression of ligands for TREM-1 and TREM-2 on tumor-infiltrating myeloid cells using TREM/Fc chimeras. Although we could not detect a TREM-1 ligand, a TREM-2 ligand was readily detectable on the surface of TAM (**Figure 3E**). These findings suggest that TREM-2 is engaged in these cells and support a role for this receptor within the tumor microenvironment.

### sTREM-1 Levels Are Increased in Tumor-Bearing Mice and Correlate With Tumor Burden

**Figure 4:**
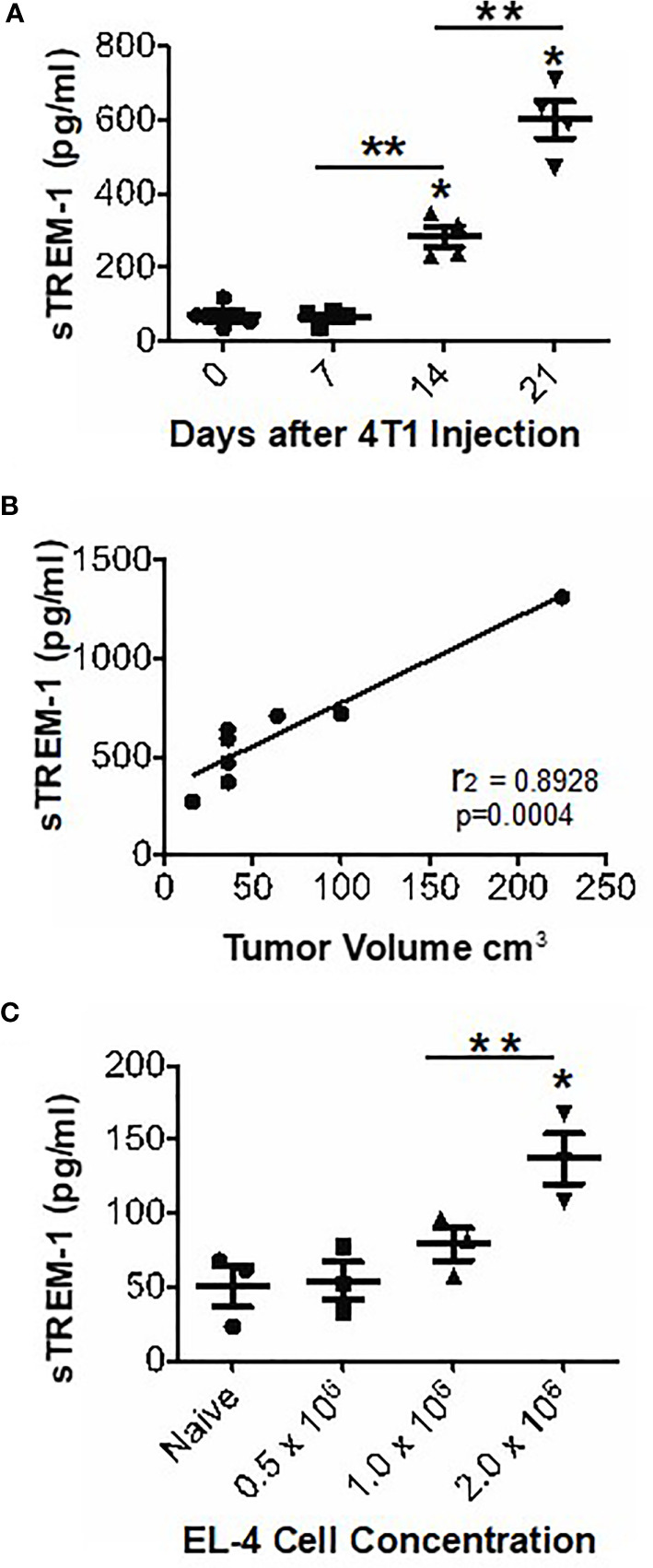
sTREM-1 levels are increased in the plasma of tumor-bearing mice and correlate with tumor burden. **(A, B)** The mice were injected with 10^4^ 4T1 cells or with varying concentrations of EL-4 thymoma cells **(C)**. The plasma sTrem-1 levels and tumor burden were assessed at various time points **(A, B)** or at 2 weeks after injection **(C)**. The sTREM-1 levels were determined by ELISA. *p-value <0.05 relative to naïve mice, **p-value <0.01 **(A, C)**. Linear regression analysis was performed to obtain an *r* ^2^ value of 0.8928 and a p-value of 0.0004 **(B)**.

Because sTREM-1 levels are associated with many inflammatory conditions (2), we predicted that sTREM-1 was increased in tumor-bearing animals. Therefore, we measured sTREM-1 levels in the plasma of naïve or 4T1-bearing mice by ELISA. The sTREM-1 levels were significantly elevated in tumor-bearing mice as early as 2 weeks following the tumor challenge and increased over time (**Figure 4A**). Direct measurement of tumors in parallel with sTREM-1 testing demonstrated that the sTREM-1 level correlated highly (*r* ^2^ = 0.8928; *p*-value = 0.0004) with tumor volume (**Figure 4B**). We verified the correlation of sTREM-1 with tumor burden using another tumor-bearing model and found that plasma sTREM-1 levels increased with an increasing concentration of EL-4 injected cells (**Figure 4C**); however, the total levels in this model were much lower than those in 4T1 even when the mice carried tumors of similar sizes, possibly due to less MDSC accumulation in EL-4 tumor-bearing mice [MDSC means of 34.83 ± 4.15 and 14.47 ± 1.07 (*n* = 3) in blood and spleen of EL-4, respectively, *vs*. 84.70 ± 5.52 and 50.17 ± 4.71 in blood and spleen of 4T1 (*p*-values <0.002 for blood and spleen)]. These findings suggest that sTREM-1 is a good early biomarker for tumor burden, possibly playing a role in tumor progression.

**Figure 5:**
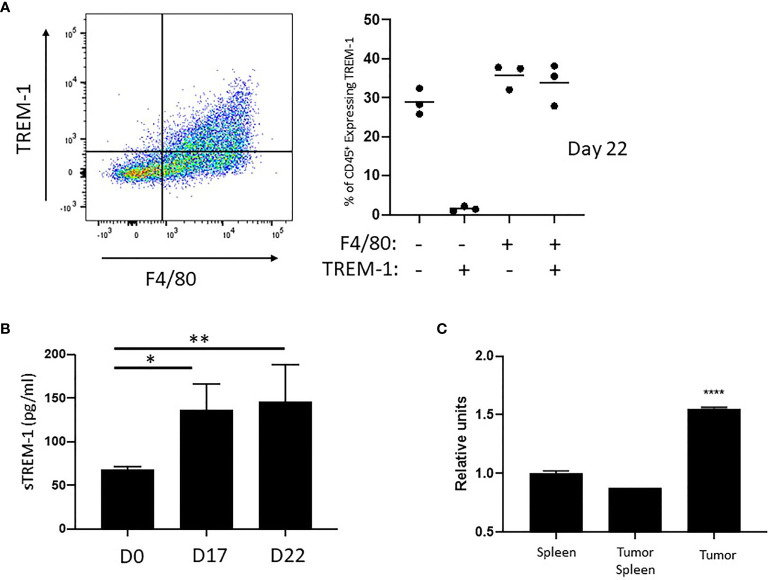
Expression of TREM in RENCA tumors. **(A)** Representative FACS plot (left) showing the staining of RENCA-associated CD45^+^ leukocytes with TREM-1 and F4/80. Data from a quadrant analysis of three individual mice are grafted (right). Staining of the quadrants is noted below the graph. **(B)** Soluble TREM-1 as determined by ELISA. The mean +/- SEM for five individual mice in each group is plotted. **(C)** qPCR analysis of *Trem2* on RNA isolated from whole RENCA tumors (tumor) at day 22 after implantation. Relative values compared to splenocytes isolated from healthy mice (spleen) and splenocytes isolated from tumor-bearing mice (tumor spleen) are shown. *p < 0.05, **p < 0.01, and ****p < 0.0001.

Although the 4T1 breast cancer model is convenient for the assessment of MDSC features due to the extreme accumulation of these cells, our laboratories have a particular interest in the peripheral response to RCC. Therefore, we investigated the possibility that TREM-1 may be expressed in the RCC model RENCA. We were able to document substantial populations of TREM-1^+^ myeloid cells within RENCA tumors (**Figure 5A**). The flow cytometry of single cell suspensions of RENCA tumors showed that nearly 70% of the recovered leukocytes expressed F4/80, and of this population, nearly half also expressed TREM-1 (**Figure 5A**). In contrast to the breast cancer model, there was little TREM-1 expression on the F4/80- population in RENCA tumors. We detected no increase in TREM-1 expression in the peripheral blood or spleen of mice harboring RENCA tumors, but similar to 4T1 and EL-4, we found significantly higher levels of sTREM-1 in the blood of mice with RCC (**Figure 5B**) and confirmed the expression of *Trem2* mRNA in RENCA tumors (**Figure 5C**). Together these data demonstrate that RCC contain infiltrating TREM-1-expressing cells and accumulate sTREM-1 in the blood.

### TREM-1^+^ Monocytes and Neutrophils Infiltrate Human Renal Tumors

**Figure 6:**
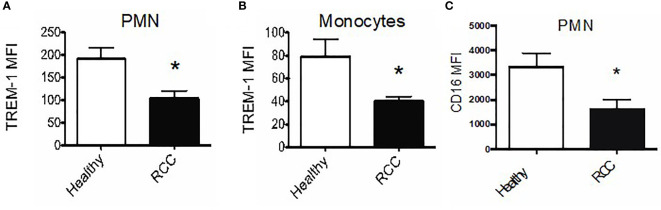
TREM-1+ myeloid cells infiltrate human renal tumors. **(A)** Average TREM-1 mean fluorescence intensity on peripheral blood polymorphonuclear neutrophils (PMN) and monocytes **(B)** from healthy (n = 3) vs. RCC donors (n = 5). **(C)** CD16 expression on peripheral blood PMN from healthy vs. RCC donors; *pvalue < 0.05.

Unlike in mice, there are no universally accepted markers for defining MDSC in humans. Therefore, we sought to assess TREM-1 expression on CD14^+^ monocytes and on neutrophils in peripheral blood and/or tumor tissues of patients with RCC (36). Intriguingly, the TREM-1 expression levels on monocytes and neutrophils of RCC donors were significantly lower than those on cells from healthy donors (40.51 ± 3.788, RCC monocytes *vs*. 79.20 ± 14.89 healthy monocytes; 103.6 ± 16.65, RCC PMN *vs*. 191.5 ± 24.52 healthy PMN) (**Figures 6A, B**). We also found decreased CD16 on PMN from RCC donors *versus* PMN from healthy donors (mean fluorescence intensity 3,368 ± 511.4, healthy *vs*. 1 647 ± 363.6, RCC, *p*-value = 0.031), suggesting that our RCC PMN were, in fact, activated (**Figure 6C**).

### sTREM-1 Expression Is Increased in RCC Patients and Correlates With Poor Outcome

**Figure 7:**
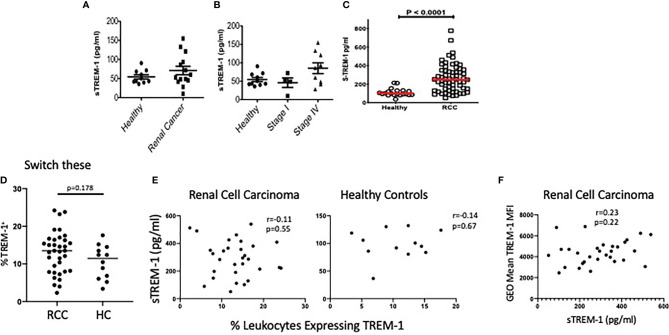
sTREM-1 levels are increased in the plasma of patients with renal cell carcinoma. **(A)** Serum from healthy donors (*n* = 10) or patients with either localized (*n* = 5) or metastatic (*n* = 9) renal cell carcinoma (RCC) was tested for soluble TREM-1 (sTREM-1) levels by ELISA. **(B)** sTREM-1 levels between stage I (localized) and stage IV (metastatic) RCC patients demonstrating a trend towards higher levels with worsening stage. **(C)** sTREM-1 levels in RCC patients from the Fox Chase Cancer Center (*n* = 63) and healthy controls (*n* = 20). **(D)** Percent of CD45+ blood leukocytes expressing TREM-1 in RCC patients and healthy controls. **(E)** Soluble TREM-1 levels are plotted *vs*. the percentages of blood leukocytes expressing TREM-1 in RCC (left) and healthy controls (right). The *r* values and *p*-values of the correlation analysis are shown. **(F)** Fluorescence intensity (geometric mean) of TREM-1 staining of RCC patients is plotted against the sTREM-1 values for those same patients. The *r* values and *p*-values of the correlation analysis are shown.

Because we observed increased sTREM-1 in RENCA tumor-bearing mice, we anticipated that the sTREM-1 levels would also be elevated in patients with RCC, a disease associated with the accumulation of aberrant neutrophils (37). Therefore, we measured the sTREM-1 levels in the plasma of a small cohort of healthy donors or RCC patients by ELISA. Indeed the sTREM-1 levels appeared to be elevated in RCC patients compared to the healthy donors (**Figure 7A**). Interestingly, the highest concentrations of sTREM-1 were found in those patients with stage IV RCC, suggesting that although the levels did not reach statistical significance, sTREM-1 could be an indicator of disease severity and/or that TREM-1 signaling might be most significant in these patients (**Figure 7B**). Based on these results, we tested a larger cohort of 63 previously untreated clear cell RCC patients accrued just prior to surgery at the Fox Chase Cancer Center in Philadelphia (**Supplementary Table S1**) that ranged in pathologic stage (stage 1, *n* = 41; stage 2, *n* = 7; stage 3, *n* = 7; and stage 4, *n* = 8) (38). These assays showed that sTREM-1 levels were, in fact, increased in patients relative to controls (mean = 265.3 pg/ml, *n* = 63 RCC *vs*. mean = 110.04 pg/ml, *n* = 20 control, *p* < 0.001)) (**Figure 7C**). A further analysis of this cohort (**Supplementary Table S1**) suggested increased sTREM-1 expression in patients that were current smokers or former smokers compared to non-smokers (*p* = 0.039) and that sTREM-1 expression in patients increases with age (*p* = 0.041). In addition, we also found significantly higher levels of sTREM-1 in patients with lymph node pathology (248.8 ± 139.0 pg/ml for patients without nodal involvement *vs*. 422.7 ± 152.7 pg/ml with nodal involvement, *p*-value = 0.005), but not with grade, overall pathologic stage, pN stage, or pM stage.

In an effort to identify the source of the sTREM-1 in RCC, we took advantage of limited flow cytometric analysis of this cohort of patients. We found no significant differences in the percentages of peripheral blood leukocytes expressing TREM-1 in RCC patients *vs*. controls (**Figure 7D**). Because our small discovery cohort suggests that peripheral cells might shed sTREM-1, we assessed the possible correlation between blood levels of sTREM-1 and the percentages of leukocytes expressing the receptor. These levels were not correlated in either healthy subjects or RCC patients (**Figure 7E**). Moreover, we found no support for the notion that sTREM-1 was being shed from the periphery, as there was no correlation between TREM-1 density, shown as a geometric mean, and the levels of sTREM-1 in the blood of RCC patients (**Figure 7F**). These data are consistent with the notion that sTREM-1 could be derived, at least in part, by the TREM-1^+^ tumor-associated leukocytes.

**Figure 8:**
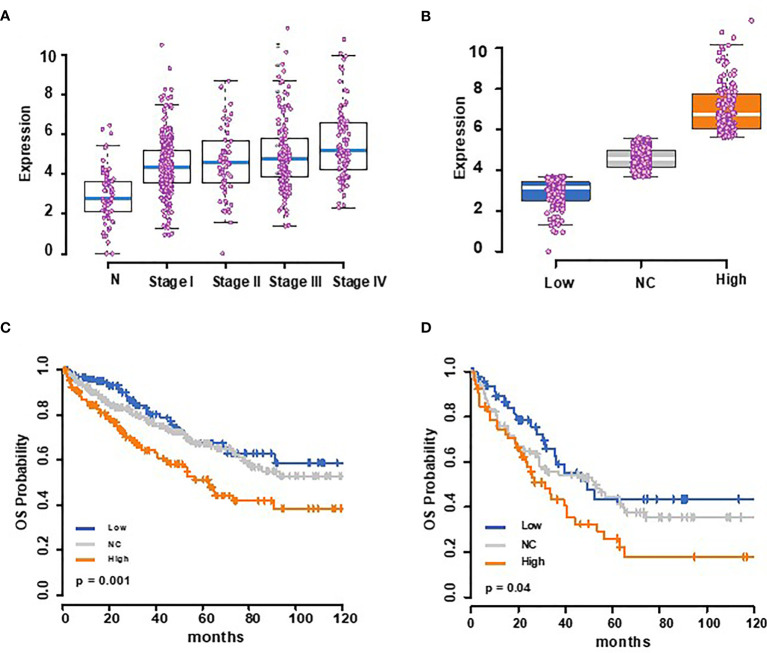
The Cancer Genome Atlas analysis shows that TREM-1 expression is increased in renal cell carcinoma (RCC) patients and correlates with a poor outcome. **(A)** Box plots showing *Trem1* mRNA expression for normal (72) and stages I–IV (*n* = 267, 57, 123, and 84, respectively) RCC patients, with the medians represented as blue bars in a box, and each case is shown as a pink dot. **(B)** *Trem1* expression levels in 531 RCC tumor samples from TCGA were stratified into tertiles of *Trem1* expression levels (low = 3rd quartile, high = 3rd tertile, and NC = intertertile range. **(C)** Kaplan–Meier overall survival curves according to this strata and **(D)** for higher-stage diseases (stage III and above).

To address this possibility more conclusively, we used mRNA expression data for 72 normal renal epithelial cells and 531 clear cell RCC tumors from the TCGA database to assess the expression changes and possible association of tumor *Trem1* to patient outcomes. The tumors showed a higher expression of *Trem1* compared to normal renal tissue (Kruskal–Wallis test *p*-value <0.001) (**Figure 8A**). Next, we stratified the 531 tumor cases into tertiles based on the RSEM gene expression values of *Trem1* and then partitioned the patients into three groups: high (upper tertile), low (lower tertile), and NC (intertertile range as no change in expression) (**Figure 8B**). When we tested whether *Trem1* expression associated with survival, we found that patients in the high tertile of *Trem1* expression had statistically worse survival when compared to those patients with low *Trem1* expression (logrank test, *p* = 0.001; HR = 2.0; 95% CI, 1.33–3.22; **Figure 8C**). When we analyzed cases that are pathological stage 3 and above, we found a similar association where a higher expression of TREM-1 portends a worse overall survival in higher-stage disease (logrank test, *p* = 0.04; HR = 1.92; 95% CI, 1.1–3.37; **Figure 8D**). Taken together these data demonstrate that patients with RCC have increased levels of sTREM-1 in their blood compared with healthy donors and that tumor-associated *Trem1* correlates with high disease stage, primary tumor size, and poor outcomes in RCC.

## Discussion

In this study, we examined the expression of the pro-inflammatory receptor TREM-1 and the anti-inflammatory receptor TREM-2 on myeloid cells in the periphery and tumors of mice and patients with cancer. In tumor-bearing mice, we found the expression of both receptors in MDSC and TAM. However, while TREM-1 was expressed in both MDSC populations, namely, Mo-MDSC and PMN-MDSC, TREM-2 was only expressed in the Mo-MDSC population. It has been previously reported that TREM-1 and TREM-2 have unique expression profiles and the dual expression of the receptors has previously been observed only on bone marrow-derived DC (11, 20, 39) and osteoclast precursors (40), although in this population TREM-1 expression negatively regulates differentiation. Most importantly, we find increased levels of soluble TREM-1 in patients with renal carcinomas, and the analysis of TCGA data shows that patient outcomes are worse than when the tumor levels of TREM-1 are highest.

Unlike murine MDSC, which are a heterogeneous mix of myeloid cells, both monocytic and granulocytic in origin, human MDSC in RCC patients have been reported to be more granulocytic (37, 41, 42). Thus, in this respect, our findings in RCC patients more closely resemble our data with murine PMN-MDSC, where we observed only slight changes in TREM-1 levels relative to neutrophils in naïve mice. In fact, our assessment of TREM-1 expression in an RCC cohort showed no differences in the percentages of cells expressing TREM-1 compared to healthy controls. While murine MDSC are immature in status, MDSC from RCC patients are frequently described as a subset of activated granulocytes (37, 41). Accordingly, we found that TREM-1 levels decrease on activated PMN (unpublished findings), and Rodriguez et al. showed that CD16 is decreased on MDSC from RCC patients, which closely resembled activated healthy PMN (37). It should be noted, however, that our data show the accumulation of sTREM-1 in the blood of RCC patients that does not correlate with any reduction in TREM-1-expressing cells. This leads us to conclude that the sTREM-1 that we find in patients may be, at least in part, derived from tumor-associated leukocytes.

Large numbers of myeloid cells, including MDSC and TAM, infiltrate solid tumors, contributing to immunosuppression (43). Indeed high numbers of tumor-infiltrating macrophages have been shown to correlate with poor patient prognosis (44). One method to overcome the immunosuppressive tumor microenvironment would be to re-direct tumor-infiltrating myeloid cells from a pro-tumor, or M2, to an anti-tumor, or M1, phenotype (45, 46). We hypothesize that myeloid cells could potentially be re-polarized through the induction of myeloid-associated receptors, such as TREMs, that, when triggered, could alter the cytokine expression profiles and thus the immunostimulatory capacity of cells. In fact, the recent work of Molgora et al. shows that TREM-2 expression supports the alternative phenotype of macrophages associated with immunosuppression and that interference with that signal yields better responses to immune checkpoint therapy (47).

Although both TREM-1 and TREM-2 signal *via* the adaptor protein DAP12, these receptors have long been known to deliver proinflammatory or anti-inflammatory signals, respectively—for example, contrary to the reported role of TREM-2 in colonic wound repair, TREM-1 has been found to promote inflammation-driven tumorigenesis (48). A plausible explanation for this dichotomy is their largely non-overlapping expression with TREM-1 primarily on neutrophils and TREM-2 on macrophages. Within the tumor site, the likely complex interactions between TREM-1-positive granulocytic cells and their TREM-2-expressing macrophages are yet to be fully dissected. Regardless, the somewhat paradoxical roles of the two receptors may be reconciled by the functions of soluble TREM-1. It is tempting to speculate that at the tumor site, as is thought to be the case during sepsis (4), sTREM-1 may be blocking the function of membrane-bound TREM-1 *via* its interference with *bona fide* TREM-1 ligands. Thus, rather than representing an activation stimulus at the tumor site, the detrimental effects of *TREM1* expression revealed by our TCGA analysis may represent a secondary method of tumor immunosuppression *via* suppression in *trans* due to soluble TREM-1. Unfortunately, the recent TREM-2 studies did not assess systemic soluble TREM-1, nor did it assess the total tumor levels of the receptors. Future work in this arena may reveal this or other unanticipated interactions.

In summary, we found that TREM-1 is highly expressed on myeloid cells in tumor-bearing mice and in patients with RCC and that TREM-2 is co-expressed with TREM-1 in MDSC and TAM. Moreover, the high expression of TREM-1 in RCC is associated with poor outcome relative to patients with low or negligible TREM-1 expression. Although more in-depth studies are needed, we suggest that manipulation of TREM-1 on MDSC and TAM may alter the function of these cells, resulting in myeloid cells with either enhanced anti-tumorigenic or pro-tumorigenic activities, respectively. Furthermore, our findings suggest that sTREM-1 may be a useful biomarker for tumor progression and that neutralization of sTREM-1 may prove effective in regulating the inflammatory response at the tumor site.

## Materials and Methods

### Mice, Cell Lines, and FACS Reagents

BALB/c and C57BL/6 mice were purchased from Charles River Laboratories (Frederick, MD) and maintained under pathogen-free conditions at the National Cancer Institute-Frederick. The experiments were performed using 8–16-week old mice in accordance with the procedures approved by the Institutional Animal Care and Use Committee. The immortalized C57BL/6 MΦ cell line (B6 line), produced as described previously (49), was maintained in complete DMEM [containing 10% fetal bovine serum (FBS), L-glutamine, and antibiotics]. The 4T1 mammary carcinoma line and the RENCA renal cell carcinoma line were purchased from ATCC (Manassas, VA), maintained in complete RPMI (RPMI-1640 supplemented with 10% FBS, L-glutamine, antibiotics, 2-ME, sodium pyruvate, non-essential amino acids, and HEPES) and passaged as described (50). The EL-4 thymoma line was purchased from ATCC, maintained in DMEM supplemented with 10% horse serum, L-glutamine, and antibiotics, and passaged according to the instructions of the manufacturer. The MC38 colon carcinoma line was maintained in RPMI-1640 supplemented with 10% FBS, L-glutamine, and antibiotics and passaged by trypsinizing, washing, and plating at low cell density. The anti-mouse FITC-F4/80, PerCP-Cy5.5-CD11b, APC-Gr-1 (RB6-8C5), and FITC-Ly6G (1A8) were bought from BD Pharmingen. The anti-mouse APC-Ly6C (1G7.10) was purchased from Miltenyi-Biotec. The anti-mouse PE-TREM-1 (174031) and PE-Rat IgG2A (54447) isotype control as well as recombinant mouse TREM-1 Fc chimera, TREM-2 Fc chimera, and human IgG1 Fc were purchased from R&D Systems. The anti-mouse FcγRII/III (2.4G2) ascites was a gift from Dr. Robert Wiltrout (NCI-Frederick). The anti-human PE-TREM-1 (TREM-26) was purchased from Biolegend. The anti-human APC-CD16 and FITC-CD14 were purchased from BD Biosciences. PE-anti-human IgG (Fc) was bought from Southern Biotech.

### Healthy and RCC Donors

Healthy volunteers were recruited either through the NCI-Frederick Research Donor Program or from volunteers at the Cleveland Clinic through an institutional review board (IRB)-approved protocol. The plasma from IRB-consented RCC patients with papillary and clear cell histology was obtained from the Cleveland Clinic. The patients had localized (*n* = 5) and metastatic (*n* = 9) diseases. Serum obtained from healthy donors and donors with clear cell and papillary RCC was analyzed for sTREM-1 levels by ELISA. The patients were grouped into stages based on the American Joint Committee on Cancer staging system (the TNM system), and sTREM-1 levels were re-plotted based on these groupings.

The sTREM-1 levels were similarly assayed in plasma obtained from blood drawn into heparinized tubes from 63 previously untreated clear cell RCC patients and 20 healthy donors, in conjunction with a previously published study at Fox Chase Cancer Center in Philadelphia. All donors provided written informed consent in accordance with HIPAA and the policies of the Fox Chase Cancer Center IRB. The grade and tumor–node–metastasis staging of patients with RCCs was based on the review by a urologic pathologist (TA-S) of surgically excised tumor, and additional patient data were obtained from the FCCC Kidney Cancer Database.

Blood from healthy donors or patients who were evaluated at the US National Cancer Institute on a Urologic Oncology Branch protocol approved by the NCI IRB gave their written informed consent for participation in the study. Samples from patients with presumed sporadic renal cell carcinoma (donor 2 was shown, at a later date, to be an inherited *SDHB* mutation carrier; Ricketts et al., in preparation) were collected into EDTA blood collection tubes. Moreover, 100 ul of whole blood was incubated with human AB serum (Sigma) to block the Fc receptors, stained with antibodies to neutrophil and monocyte antigens, fixed with BD Fixative, and analyzed using a FACSort flow cytometer (BD Biosciences). For analysis of a larger RCC cohort, peripheral blood was stained and TREM-1 percentages were calculated by gating on leukocytes by scatter followed by singlet discrimination, live/dead discrimination with propidium iodide, and finally gating on TREM-1-positive cells. The percentages of leukocytes and TREM-1 fluorescence intensity (geometric mean of positive cells) were calculated using FlowJo software.

For the isolation of tumor-infiltrating leukocytes, tumors from patients with presumed sporadic RCC carcinoma (donor 2 was shown, at a later date, to be an inherited *SDHB* mutation carrier; Ricketts *et al.*, in preparation) were minced into a paste-like solution using iris surgical scissors, suspended in 20 ml RPMI containing 10% FCS, 200 U/ml collagenase IV, and 100 U/ml DNAse I, and incubated with shaking for 15 min. The cells were washed with cold Hank’s balanced salt solution (HBSS) containing 0.1% bovine serum albumin (BSA) and 0.5 mM EDTA, resuspended in 40% Percoll, and underlaid with 80% Percoll. The gradients were centrifuged at room temperature for 25 min at 850*g*. The leukocytes were collected at the interface, washed, and counted using a Sysmex KX-21 (Roche, Indianapolis, IN) automated cell counter. For flow cytometry, Fc receptors were blocked, and 1 to 2 × 10^6^ cells were stained and analyzed as indicated above.

### Transplantation of Tumors in Mice

4T1 cells (1 × 10^4^–1 × 10^6^) were injected s.c. as described (50). EL-4 (0.5 × 10^6^–2 × 10^6^) or MC38 cells (0.5 × 10^6^–1 × 10^6^) were injected s.c. into the flank of C57BL/6 mice. For RENCA tumors, 1 × 10^5^ RENCA cells were injected s.c. in 100 ul of sterile saline. The tumors were excised and digested either manually or enzymatically. For enzymatic digestion of 4T1, EL-4, and MC38, the tumors were minced in six-well plates containing a digestion cocktail (RPMI-1640 supplemented with 0.5% FBS, 100 μg/ml DNase I, and 200 U/ml Collagenase IV) and incubated at 37°C for 1 to 2 h with gentle shaking every 30 min. The tumor cells were washed with 0.5× HBSS containing 5 mM EDTA, and single-cell suspensions were prepared by manually digesting the remaining tumor cell clumps and filtering over a 100-μm nylon mesh cell strainer. The single-cell suspensions were washed with HBSS containing 5 mM EDTA. For cell preparation from RENCA, the tumors were homogenized using a GentleMACS Tissue Homogenyzer (Mitenyi Biotec) in RPMI media supplemented with 15 mg collagenase IV (Sigma), 0.5 mg/ml DNase I (Sigma), and 5% fetal bovine serum. The homogenized tissues were incubated at 37°C for 30 min and filtered through a 70-uM nylon filter. The red blood cells were lysed using ACK buffer (Invitrogen). The cells were incubated with fluorescently conjugated antibodies or isotype controls and analyzed by flow cytometry. Flow cytometric analysis of infiltrating populations followed the gating schemes in **Supplementary Figures S1** and **S2** with the addition of CD45 gating prior to lineage gating.

### Cell Sorting

Single-cell suspensions of splenocytes or tumor cells were prepared by manual or enzymatic digestion, respectively. The red blood cells were lysed using ACK lysis buffer. Then, 400 × 10^6^ splenocytes or 100 × 10^6^– 400 × 10^6^ tumor cells were washed and resuspended in PBS containing 0.1% BSA or 2% BSA and 4 mM EDTA, respectively. The Fc receptors were blocked with 2.4G2, and splenocytes or tumor cells were stained and sorted using BD FACSAria. Gating for sorting was as shown in **Supplementary Figure S2**. The purity of sorted populations was confirmed to be >99% by analysis of post-sorted populations.

### T Cell Proliferation Assays

Sorted MDSC were incubated in 0.2 ml complete RPMI in 96-well U-bottom plates with syngeneic splenocytes (5 × 10^4^/well) at the indicated ratios. Con A was added to each well at a final concentration of 2 μg/ml. Tritiated thymidine (1 μCi/well) incorporation was measured during the last 18 h of the 85-h incubation. The plates were harvested onto 96-well filter mats, and counts were measured with a Wallace microbeta counter (Perkin Elmer).

### Stimulation of MDSC With Anti-TREM-1

The PMN-MDSC were sorted from spleens of 4T1 tumor-bearing mice as described above. Then, 10 μg/ml anti-TREM-1 (174031) or rat IgG2A isotype control (both from R&D) was coated onto the wells of a 96-well flat-bottom plate in 0.1 ml PBS. At 3 h later, the plates were washed with PBS, and LPS was added at a concentration of 10 μg/ml in complete DMEM. A total of 10^5^ MDSC were added to each well (final well volume of 0.2 ml), and the plates were spun for 3 min to engage plate-bound Ab. The supernatants were harvested 20 h later.

### ELISA

The mouse TREM-1, human TREM-1, and mouse TNF quantikine ELISA kits were purchased from R&D. For sTREM-2 detection, Costar 96-well half-area assay plates were coated with 3 μg/ml rat anti-mouse TREM-2 (clone 237920) in borate-buffered saline. TREM-2/Fc chimera (R&D) was used to generate a standard curve, and TREM-2 was detected using biotinylated sheep-anti-mouse TREM-2 (R&D) followed by streptavidin–horseradish peroxidase (Southern Biotech). The plates were developed using BD OptEIA TMB substrate reagent set and stopped by adding an equal volume of 0.18 M sulfuric acid to each well. The plates were read using a Versamax microplate reader with SoftMax Pro software (Molecular Devices). We confirmed the specificity of our TREM-2 ELISA by measuring the supernatants from 293 cells transfected with plasmids encoding the extracellular domains of TREM-1 or TREM-2.

### Quantitative RT-PCR

RNA was isolated from cell lines or sorted MDSC and TAM populations using Trizol reagent (Invitrogen) in combination with QIAGEN RNeasy kit according to the protocol of the manufacturer. cDNA was generated using TaqMan Reverse Transcription Reagents (Applied Biosystems). *Trem2* message was quantitated from cDNA using mouse TREM-2 TaqMan gene expression assay (Mn00451744_m1) and TaqMan Universal PCR Master Mix (both from Applied Biosystems). cDNA synthesis and quantitative PCR were performed according to the protocol of the manufacturer. Real-time PCR reactions were run in 96-well plates on ABI 7300 (Applied Biosystems). HPRT was used as the housekeeping gene.

### Statistics

Unless otherwise noted, the *p*-values were determined by Student’s unpaired *t*-test in Prism 5 (GraphPad Software, La Jolla, CA). The *p*-values <0.05 were considered significant. Correlations between patient TREM-1+ populations and sTREM and/or means of TREM-1 expression were calculated using Prism using two-tailed tests. Differences in sTREM-1 expression by patient characteristics were determined using analysis of variance for nominal variables (gender, race, smoking history, pN, pM, and histology), where *p*-values from F-statistics are reported. A linear trend in sTREM-1 expression was assessed for ordered variables (age, grade, pathologic stage, and pT) using linear regression. For the TCGA mRNA expression and survival analyses, TREM-1 expression data was obtained as RSEM gene expression values (log2-transformed), and overall survival outcomes obtained from the Broad Firehose pipeline were considered for analysis. The RSEM values were categorized into three categories as tertiles. Kruskal–Wallis test was applied to compare the expression differences between normal and different tumor stages. The Kaplan–Meier overall survival curves were compared with logrank tests and were generated using the survival package in R computing environment. Cox regression models were applied on the overall survival prediction between the three expression categories.

## Data Availability Statement

The raw data supporting the conclusions of this article will be made available by the authors, without undue reservation.

## Ethics Statement

The studies involving human participants were reviewed and approved by the NCI-Frederick Research Donor Program or Cleveland Clinic. The patients/participants provided their written informed consent to participate in this study. The animal study was reviewed and approved by the Institutional Animal Care and Use Committee, National Cancer Institute-Frederick.

## Author Contributions

All authors contributed to the study conception and design. The experiments and data collection were performed by JWF, JS, and OH. Statistical and data mining was performed by AM, TA-S, PR, YY, and JF. Human sample collection and analysis were carried out by SP, KR, MH, TA-S, BR, and WL. Manuscript preparation, data analysis, and editing were performed by JWF, MG-C, KC, and DM. All authors contributed to the article and approved the submitted version.

## Funding

This project has been funded in whole or in part with federal funds from the National Cancer Institute, National Institutes of Health, under contract HSN261200800001E. The content of this publication does not necessarily reflect the views or policies of the Department of Health and Human Services nor does the mention of trade names, commercial products, or organizations imply endorsement by the US Government. This Research was supported (in part) by the Intramural Research Program of the Center for Cancer Research, National Cancer Institute, National Institutes of Health. Additional support was provided by the Personalized Kidney Cancer Therapy Keystone Program at Fox Chase Cancer Center (FCCC) and NCI Comprehensive Cancer Center Support Grant CA06927 (FCCC).

## Conflict of Interest

Author BR: Research Funding to Institution: Pfizer, Hoffman-LaRoche, Incyte, AstraZeneca, Seattle Genetics, Arrowhead Pharmaceuticals, Immunomedics, BMS, Mirati Therapeutics, Merck, Surface Oncology, Dragonfly Therapeutics, Aravive, Exelixis, Jannsen. Consulting: BMS, Pfizer, GNE/Roche, Aveo, Synthorx, Compugen, Merck, Corvus, Surface Oncology, 3DMedicines, Aravive, Alkermes, Arrowhead, GSK, Shionogi, Eisai, Nikang Therapeutics. Stock: PTC therapeutics.

The remaining authors declare that the research was conducted in the absence of any commercial or financial relationships that could be construed as a potential conflict of interest.

## Publisher’s Note

All claims expressed in this article are solely those of the authors and do not necessarily represent those of their affiliated organizations, or those of the publisher, the editors and the reviewers. Any product that may be evaluated in this article, or claim that may be made by its manufacturer, is not guaranteed or endorsed by the publisher.

## Abbreviations

TREM, triggering receptors expressed on myeloid cells; TLT, TREM-like transcript; sTREM, soluble TREM; CSF, cerebrospinal fluid; MDSC, myeloid-derived suppressor cells; PMN-MDSC, granulocytic MDSC; Mo-MDSC, monocytic MDSC; TAM, tumor-associated macrophages; RCC, renal cell carcinoma.
