# Supplementary figures and images for: Tumor-Infiltrating Myeloid Cells Co-Express TREM1 and TREM2 and Elevated TREM-1 Associates With Disease Progression in Renal Cell Carcinoma
Source: Front Oncol. 2022 Feb 10;11:662723. doi: 10.3389/fonc.2021.662723 (PMC8867210; doi:10.3389/fonc.2021.662723)

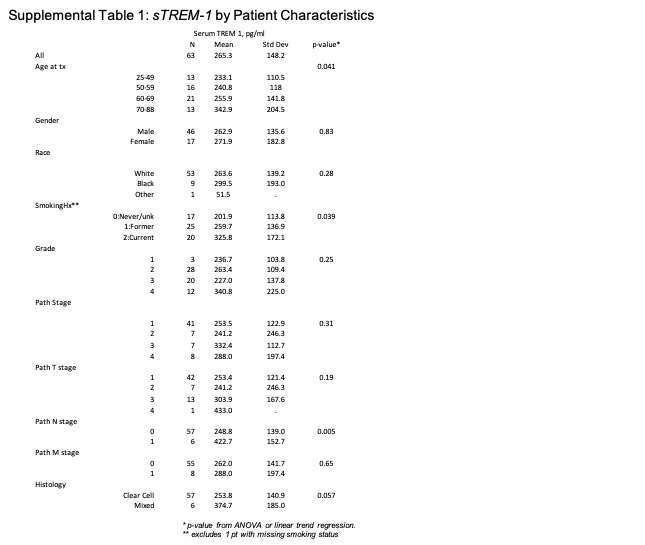

Supplement: Supplementary file 1 [file DataSheet_1.zip › Supplementary Table 1.JPEG]

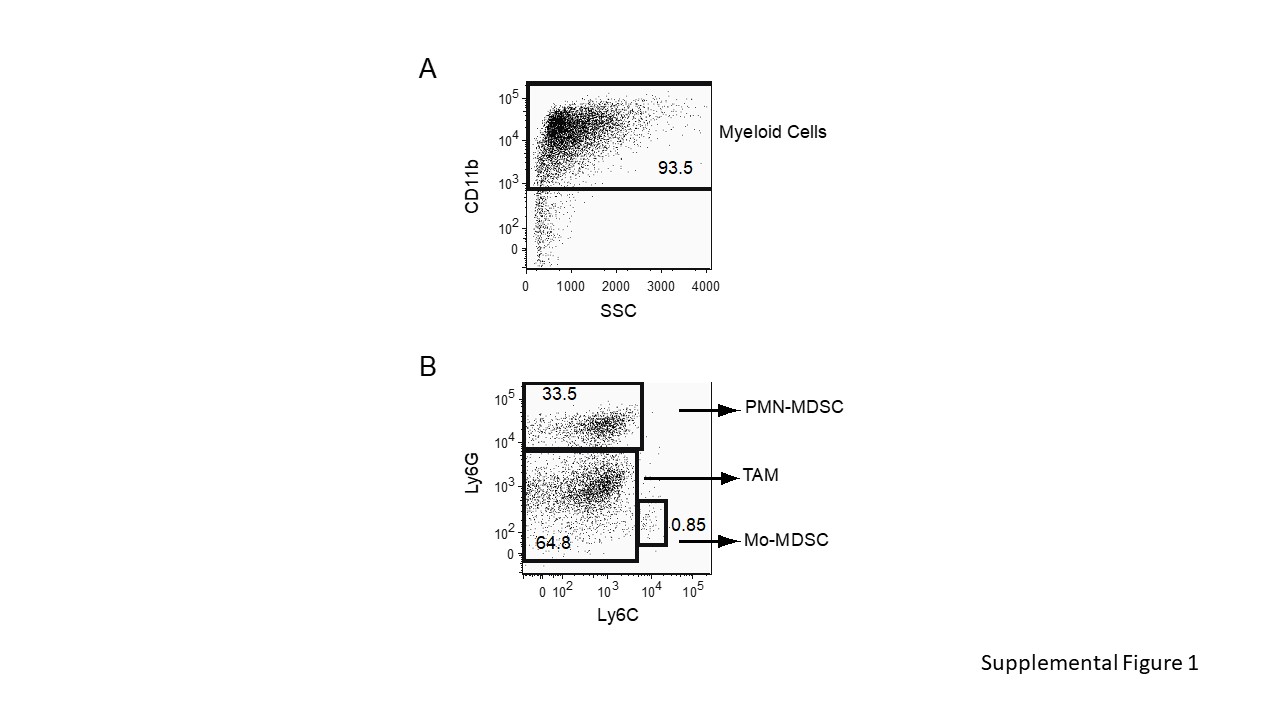

Supplement: Supplementary file 2 [file Image_1.jpg]

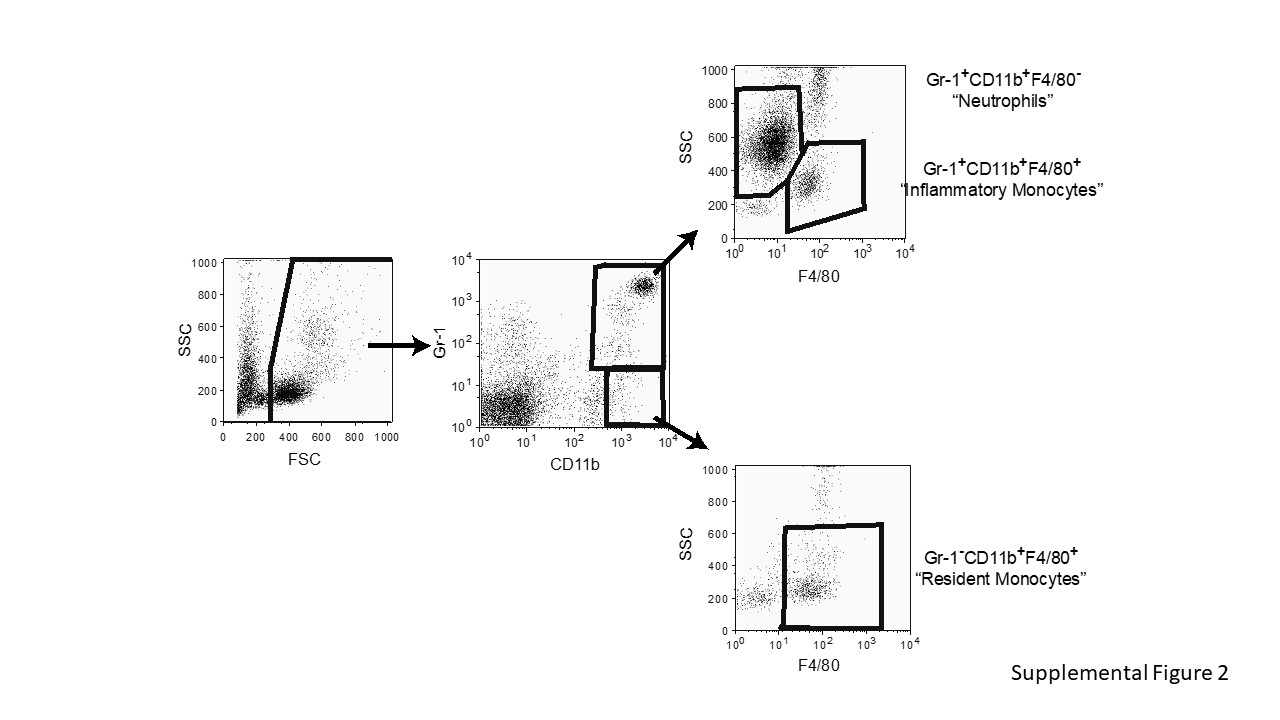

Supplement: Supplementary file 3 [file Image_2.jpg]

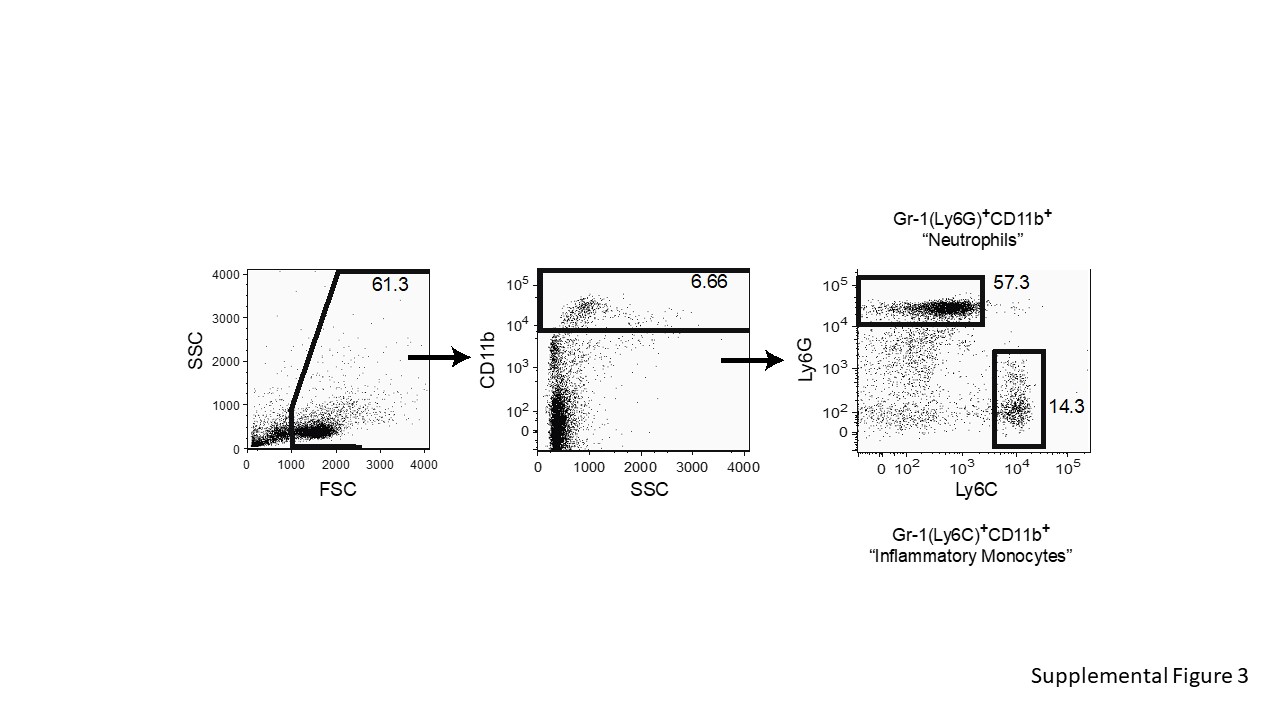

Supplement: Supplementary file 4 [file Image_3.jpg]

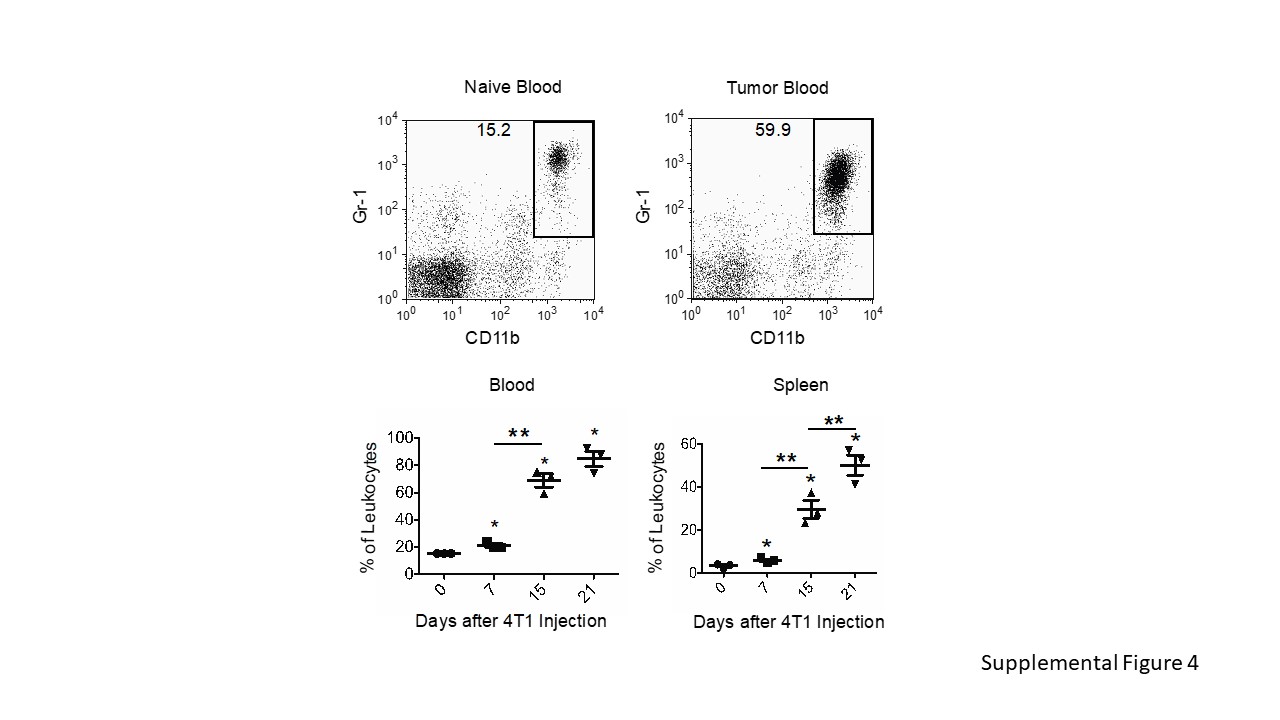

Supplement: Supplementary file 5 [file Image_4.jpg]
